# Unusual presentation of Castleman's disease mimicking lung cancer

**DOI:** 10.1002/rcr2.416

**Published:** 2019-03-14

**Authors:** Ming‐Tsung Chen, Shih‐Chun Lee, Chun‐Chi Lu, Chen‐Liang Tsai

**Affiliations:** ^1^ Division of Pulmonary and Critical Care, Department of Internal Medicine Tri‐Service General Hospital, National Defense Medical Center Taipei Taiwan; ^2^ Division of Thoracic Surgery, Department of Surgery Tri‐Service General Hospital, National Defense Medical Center Taipei Taiwan; ^3^ Division of Rheumatology/Immunology/Allergy, Department of Internal Medicine Tri‐Service General Hospital, National Defense Medical Center Taipei Taiwan

**Keywords:** Castleman's disease, lymphadenopathy, peribronchovascular interstitial thickening

## Abstract

Castleman's disease (CD) is an uncommon lymphoproliferative disorder characterized as either unicentric or multicentric presentation based on the involving sites. The most frequent presentation of CD is a solitary mediastinal mass. We reported a patient with a history of heavy smoking with particular image features of CD, which presented as mediastinal lymphadenopathy and peribronchovascular interstitial thickening mimicking lung cancer or sarcoidosis initially.

## Introduction

Castleman's disease (CD) is a rare heterogeneous group of lymphoproliferative disorders characterized by angiofollicular lymph node hypertrophy and first reported in 1954 by Benjamin Castleman who described a 40‐year‐old male with a mediastinal mass characterized histologically by hyperplastic lymph node with regressed germinal center. According to the lesions involved, CD is categorized as unicentric (UCD) or multicentric (MCD), and approximately 70% of patients present the disease in the thorax. The most common manifestation of CD is a solitary mediastinal mass, and rarely parenchymal pulmonary involvement may be seen 1, 2. Here we report the case of a patient with a history of heavy smoking with initial clinical suspicion of pulmonary malignancy but eventually diagnosed with CD.

## Case Report

**Figure 1:**
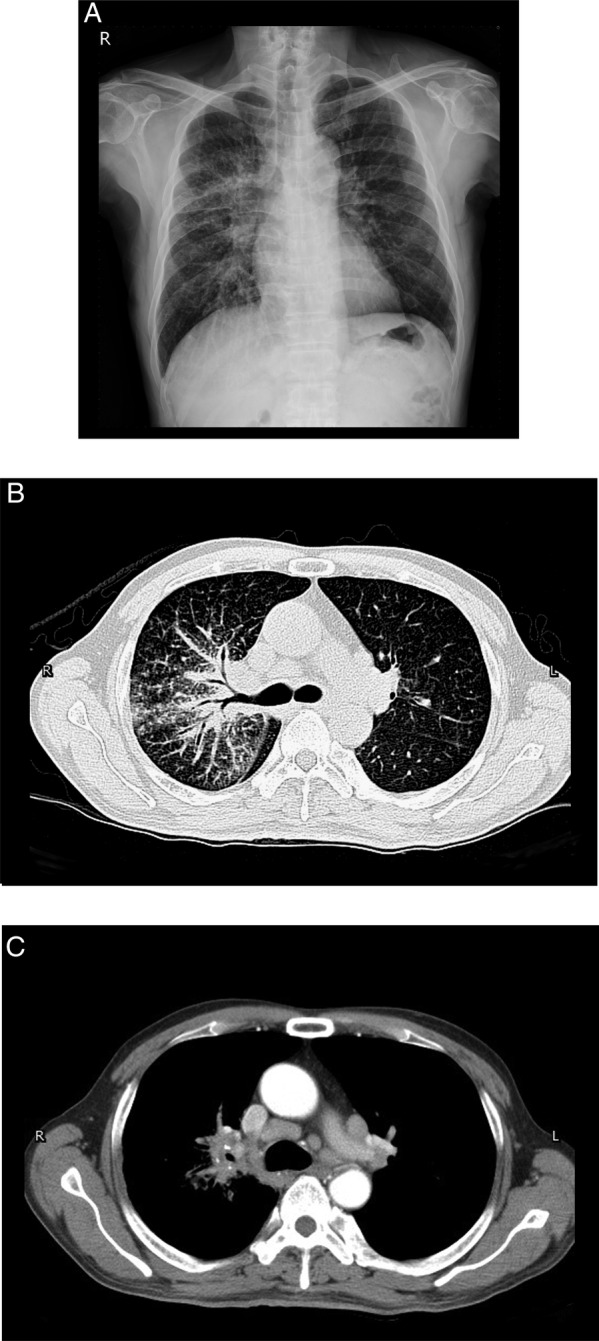
(A) Chest X‐ray showed increased interstitial marking right site, and patchy consolidations of the right lung. (B) Chest computed tomography (CT) showed unilateral small centrilobular nodules, and smooth peribronchovascular interstitial thickening, particularly in the right lung. (C) Contrast‐enhanced chest CT showed multiple enlarged mediastinal lymph nodes.

**Figure 2:**
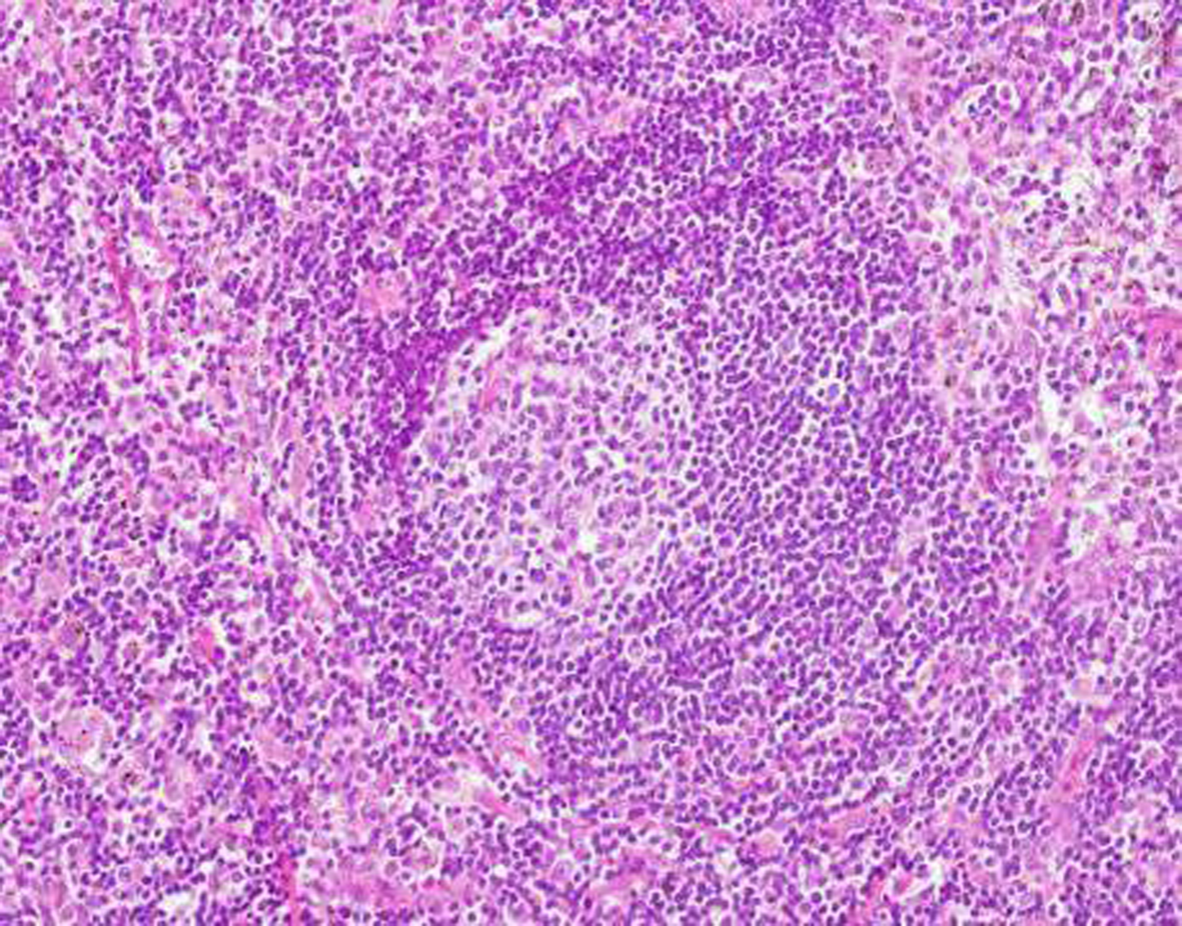
Histological findings of the excisional mediastinal lymph node showed lymphoid follicle proliferation characterized by concentric rings of small lymphocytes and sclerotic blood vessels radially penetrating the germinal centers.

**Figure 3:**
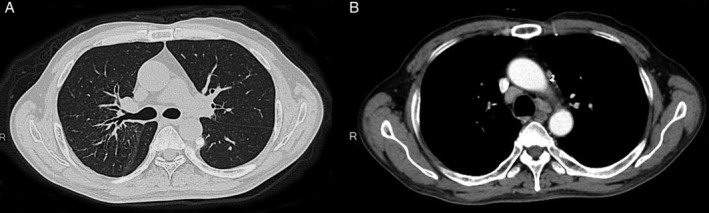
After treatment, (A) chest computed tomography (CT) and (B) contrast‐enhanced chest CT showed significantly regressive change.

A 56‐year‐old male, without previous systemic disease, presented with body weight loss about 10 kg and dry cough for about two months. He was a heavy smoker with a 40 pack‐year history of smoking. Physical examination findings were unremarkable. The chest X‐ray showed increased interstitial marking on the right side, and patchy consolidations of the right lung (Fig. 1A). A chest computed tomography (CT) revealed multiple enlarged mediastinal lymph nodes, unilateral small centrilobular nodules, and smooth peribronchovascular interstitial thickening without traction bronchiectasis particularity in the right lung (Fig. 1B,C). The differential diagnosis included lung cancer with nodal metastasis and sarcoidosis. The tumour markers were within normal range, and the bronchoscopy showed no endobronchial lesion. In autoimmune biomarkers survey, the only abnormality was the elevation of rheumatoid factor immunoglobulin M (14.5 IU/mL). However, due to the clinical suspicion of malignancy and interstitial lung disease, adequate tissue for diagnosis was crucial. We suggested a parasternotomy approach (Chamberlain procedure) with mediastinal lymph node excision. The pathology of the mediastinal lymph node showed lymphoid follicle proliferation characterized by concentric rings of small lymphocytes and sclerotic blood vessels radially penetrating the germinal centers (Fig. 2). Immunohistochemical staining for human herpes virus‐8 (HHV‐8) was negative. The picture was compatible with hyaline vascular type CD. Screen of blood HHV‐8 and human immunodeficiency virus (HIV) test were negative. Then corticosteroid and tocilizumab were prescribed, and the following chest CT showed completely regressive change with significant clinical improvement (Fig. 3).

## Discussion

CD is an exceptional lymphoproliferative disorder striking single (UCD) or multiple (MCD) lymph nodes. Extranodal or pulmonary parenchymal involvement is not a frequent presentation in CD. The typical CT appearance of the intrathoracic hyaline vascular CD is an avidly enhancing mediastinal nodal mass and mimics lymphoma, thymoma, sarcoma, or neurogenic tumour. There is no distinct image presentation on chest CT. One retrospective observational study collected data of 22 intrathoracic CD patients with pulmonary parenchymal involvement, and revealed multiple different size nodules (72.7%), cysts (59.1%), ground glass opacity (54.5%), consolidation (22.7%), thickening of interlobular septum (36.4%), and bronchovascular bundles (31.8%) 1. In our case, bronchogenic carcinoma with nodal involvement or sarcoidosis was the first suspicion. However, typical lymphangitic carcinomatosis features, including nodular or beaded interstitial thickening, reticular opacities, and pleural effusion, were not observed. At the same time, absent bilateral pulmonary invasion, fibrosis, and symmetric bilateral hilar lymphadenopathy were weakening the sarcoidosis diagnosis 3. In recent years, immunoglobulin G4 (IgG4)‐related disease attracted attention and also involved multiple organs characterized by dense lymphoplasmacytic infiltrations with abundant IgG4‐positive plasma cells. The pulmonary manifestations of IgG4‐related disease included mediastinal lymphadenopathy, multiple ground glass opacities, a mass, bronchiectasis, and bronchovascular bundles thickening 4. To better obtain sufficient tissue for diagnosis and reduce harm to the patient, we chose the Chamberlain procedure approach instead of endobronchial ultrasound‐guided transbronchial needle aspiration (EBUS‐TBNA) to obtain enough tissue. EBUS‐TBNA is a useful tool used to take samples of mediastinal lesions without an open wound, but insufficient sample volume frequently prevents the pathologist from making a diagnosis.

It is challenging to make the diagnosis of CD simply using image features. Comprehensive studies, including pathology and serology, are inevitable. There are three histopathological types: hyaline‐vascular, plasma cell type, and mixed type. Approximately 90% CD is the hyaline‐vascular type, which is the most common feature of UCD. Hyaline‐vascular type CD occurred most often in young adults and diagnosed in the third or fourth decade. However, plasma cell type typically presents in MCD and frequently associated with systemic manifestations, such as fever, malaise, and hematologic abnormality 5, 6. Confirming the diagnosis of CD is a challenge, and satisfactory biopsy of involved tissue is necessary.

UCD is treated by complete surgical resection, which allows full recovery without relapse in almost all cases, and radiotherapy is a choice in unresectable UCD. MCD might be related to HHV‐8 infection, which is highly co‐infected with HIV, and the initial therapeutic strategy is distinguishing whether virus infection is present or not. Many systemic treatment choices, including chemotherapy, corticosteroid, anti‐CD‐20 antibody, and anti‐interleukin‐6 (IL‐6) receptor are used in HHV‐8‐negative CD 6, 7. In our case, we prescribed corticosteroid and anti‐IL‐6 therapy (tocilizumab), and then successfully achieved disease remission and symptomatic relief.

Our case highlights the point that, in patients with an initial presentation of mediastinal lymphadenopathy and lung parenchymal involvement, physicians should keep in mind the possibility of CD as differential diagnosis for malignancy. Also, CD is a pathological diagnosis, and adequate lymph node specimen is essential for definite diagnosis.

### Disclosure Statement

Appropriate written informed consent was obtained for publication of this case report and accompanying images.

## References

[1] Huang H , Feng R , Li J , et al. 2017 Castleman disease‐associated diffuse parenchymal lung disease: a STROBE‐compliant retrospective observational analysis of 22 cases in a tertiary Chinese hospital. Medicine 96:e8173. 28953671 10.1097/MD.0000000000008173 PMC5626314

[2] Johkoh T , Muller NL , Ichikado K , et al. 1998 Intrathoracic multicentric Castleman disease: CT findings in 12 patients. Radiology 209:477–481. 9807577 10.1148/radiology.209.2.9807577

[3] Castaner E , Gallardo X , Pallardo Y , et al. 2005 Diseases affecting the peribronchovascular interstitium: CT findings and pathologic correlation. Curr. Probl. Diagn. Radiol. 34:63–75. 15753880 10.1067/j.cpradiol.2004.12.002

[4] Campbell SN , Rubio E , and Loschner AL . 2014 Clinical review of pulmonary manifestations of IgG4‐related disease. Ann. Am. Thorac. Soc. 11:1466–1475. 25422997 10.1513/AnnalsATS.201403-128FR

[5] Bonekamp D , Horton KM , Hruban RH , et al. 2011 Castleman disease: the great mimic. Radiographics 31:1793–1807. 21997995 10.1148/rg.316115502

[6] Soumerai JD , Sohani AR , and Abramson JS . 2014 Diagnosis and management of Castleman disease. Cancer Control 21:266–278. 25310208 10.1177/107327481402100403

[7] Chan K‐L , Lade S , Prince HM , et al. 2016 Update and new approaches in the treatment of Castleman disease. J. Blood Med. 7:145–158. 27536166 10.2147/JBM.S60514 PMC4976903

